# *SCT*: a suite of programs for comparing atomistic models with small-angle scattering data

**DOI:** 10.1107/S1600576715007062

**Published:** 2015-05-09

**Authors:** David W. Wright, Stephen J. Perkins

**Affiliations:** a Department of Structural and Molecular Biology, Division of Biosciences, University College London, Darwin Building, Gower Street, London WC1E 6BT, UK

**Keywords:** analytical ultracentrifugation, constrained modelling, hydration shells, neutron scattering, X-ray scattering

## Abstract

Small-angle X-ray and neutron scattering techniques characterize proteins in solution and complement high-resolution structural studies. They are of particular utility when large proteins cannot be crystallized or when the structure is altered by solution conditions. Atomistic models of the averaged structure can be generated through constrained modelling, a technique in which known domain or subunit structures are combined with linker models to produce candidate global conformations. By randomizing the configuration adopted by the different elements of the model, thousands of candidate structures are produced. Next, theoretical scattering curves are generated for each model for trial-and-error fits to the experimental data. From these, a small family of best-fit models is identified. In order to facilitate both the computation of theoretical scattering curves from atomistic models and their comparison with experiment, the *SCT* suite of tools was developed. *SCT* also includes programs that provide sequence-based estimates of protein volume (either incorporating hydration or not) and add a hydration layer to models for X-ray scattering modelling. The original *SCT* software, written in Fortran, resulted in the first atomistic scattering structures to be deposited in the Protein Data Bank, and 77 structures for antibodies, complement proteins and anionic oligosaccharides were determined between 1998 and 2014. For the first time, this software is publicly available, alongside an easier-to-use reimplementation of the same algorithms in Python. Both versions of *SCT* have been released as open-source software under the Apache 2 license and are available for download from https://github.com/dww100/sct.

## Abstract 2

The *SCT* suite of tools for the atomistic modelling of X-ray and neutron scattering curves has resulted in 77 macromolecular structures to date. *SCT* is now made publicly available as open source, alongside an easier-to-use reimplementation of the same algorithms in Python.

## 1. Introduction   

Small-angle X-ray and neutron scattering (abbreviated as SAXS and SANS, and collectively as SAS) are diffraction techniques used to investigate the structural properties of condensed matter systems, including proteins, metal alloys, colloids and synthetic polymers in bulk or in solution (Perkins *et al.*, 2008 ▶, 2011 ▶; Blanchet & Svergun, 2013 ▶). SAS is widely used to study biological macromolecules in solution, in particular to characterize large proteins that cannot be crystallized or where solution conditions affect the structure. The advantage of working in near physiological solution conditions with SAS is counterbalanced by the lower resolution of the structural information compared to techniques such as crystallography. In the absence of crystalline order in SAS, the results are necessarily averaged over the orientations and conformations occupied in solution. Consequently, SAS is generally viewed as a complement to higher-resolution techniques. Structural information from SAXS/SANS data is extracted by the use of curve fitting for general shape metrics (such as Guinier fits for the radius of gyration, *R*_g_), *ab initio* shape determination and rigid-body refinement (Svergun & Koch, 2003 ▶), or constrained modelling based on known crystal structures (Perkins *et al.*, 2011 ▶). In this paper we describe a software suite, *SCT*, designed to facilitate the constrained scattering modelling of protein, glycoprotein and carbohydrate systems (Perkins *et al.*, 2011 ▶).

Constrained modelling is based on the comparison of the theoretical scattering curves generated from conformationally randomized trial atomistic models of the target protein with those obtained from experiment. Known structures for the domains or subunits (such as crystal structures) are combined with linker models that join these to produce candidate global conformations. By randomizing the global conformation, thousands of candidate structures can be produced and their theoretical scattering curves calculated. These curves are used for trial-and-error fits to the experimental data in order to identify a family of best-fit models. It should be noted that fitting detailed models to low-resolution data is an under-determined problem. No unique solution is available, and the end result represents the average structure. Nonetheless, the method is effective in rejecting structures that are not compatible with the scattering data. The *SCT* suite was developed for both the computation of theoretical scattering curves from atomistic models and their comparison with experiment. The original Fortran *SCT* software, which we will refer to as the ‘classic’ version, has been used to determine 77 structures (24 antibodies, 27 complement proteins and 24 oligosaccharides) deposited in the Protein Data Bank (PDB; http://www.pdb.org/) between 1998 and 2014 (Supplementary Table S1^1^). Here we describe the classic version of *SCT* for the first time and make this publicly available, alongside a new easier-to-use reimplementation of the same algorithms in Python (this version will be referred to as the ‘modern’ version). Both versions of *SCT* are available as open-source software from https://github.com/dww100/sct. Version 1.0.0 of SCT is also archived in Zenodo at the CERN data centre for long-term accessibility and storage (http://dx.doi.org/10.5281/zenodo.16083).

## 2. Background to small-angle scattering and constrained modelling   

**Figure 1:**
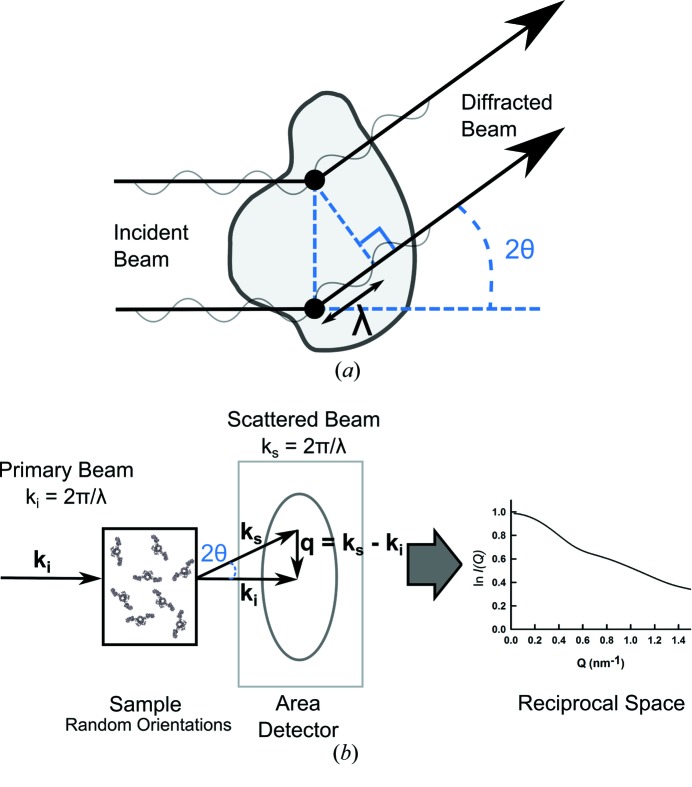
Schematic representation of a scattering experiment. (*a*) An incident beam is shown scattering from two point scatterers (represented by the black dots) within a globular macromolecule. The diffracted rays are in phase with each other but out of step by λ at the scattering angle 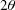 shown, causing constructive interference. The accumulation of these events at low angles gives rise to the scattering pattern of the macromolecule. (*b*) In a typical small-angle scattering experiment, diffraction from high-scattering-density macromolecules in a low-scattering-density solution gives rise to a scattering pattern on an area detector. **q** is the scattering vector 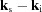. The radial average of the scattering pattern about the position of the direct main beam gives rise to the scattering curve 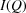 in reciprocal space.

In order to explain constrained modelling, it is necessary to outline the SAS experiments that generate the data to be modelled. Whilst there are fundamental differences in the elastic scattering of X-rays and neutrons, these processes can be described using the same mathematical framework (Glatter & Kratky, 1982 ▶). Fig. 1 ▶(*a*) shows the scattering of an incident beam by two point scatterers within a globular macromolecule. The diffracted rays are in phase with one another but out of step by 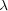 at a scattering angle of 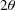, resulting in constructive interference. The accumulation of these events at low angle gives rise to the scattering curve. The important difference between SAXS and SANS is that X-ray scattering involves diffraction events from electrons, whereas neutrons are scattered by atomic nuclei. The significance of this difference for constrained modelling is that a hydration layer of water surrounding the target protein is ‘visible’ in SAXS but not for SANS experiments performed in ^2^H_2_O buffers (Perkins, 2001 ▶).

The basic setup of a SAS experiment involves irradiation of a sample by a monochromatic beam of X-rays or neutrons (Glatter & Kratky, 1982 ▶; Perkins *et al.*, 2011 ▶; Blanchet & Svergun, 2013 ▶). Differences in the scattering density contrast between solute and solvent give rise to diffraction (Fig. 1 ▶ *b*). The scattering is characterized by the scattering vector **q** (Fig. 1 ▶ *b*), the magnitude of which is given by 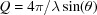, where 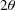 is the scattering angle and 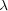 the wavelength. Small angles correspond to low *Q* values. If the sample is idealized as a dilute solution of monodisperse, non-interacting, identical particles, the experiment results in the radially averaged scattered intensity 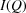 as a function of *Q*. The 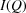 curve reduces sharply as *Q* increases. 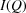 can be interpreted as a Fourier transform (reciprocal space) representation of the distance distribution of the point scatterers within the system of interest. Typical experimental *Q* ranges extend from 0.05 to 2 nm^−1^ and correspond to a real-space resolution of approximately 2–4 nm if no other constraints are applied.

Guinier analyses of the experimental scattering curves involve a linear fit of the low-*Q* region of the 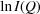 curve against 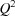 to determine 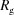 and the forward scattered intensity at zero, 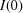:
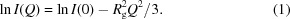
The approximation in the Guinier fit requires that the 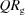 values in the fit range are between approximately 0.5 and 1.5. For elongated macromolecules, the mean cross-sectional radius of gyration 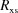 is determined using fits in a larger *Q* range that does not overlap with that used for the 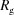 determinations [see equation (2)]:
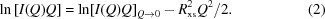
These analyses can be applied just as easily to theoretically generated curves as those from experiment.

**Figure 2:**
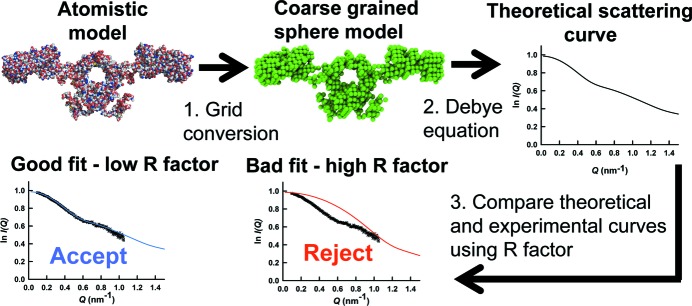
Constrained modelling algorithm in *SCT*. First, candidate full atomistic structures of the target macromolecule are generated. That illustrated is for human IgA1, taken from Boehm *et al.* (1999 ▶). A grid transformation is performed on each structure to produce a lower-resolution (coarse-grained) sphere model, which is used to calculate a theoretical scattering curve *via* the Debye equation. The *R* factor determines if the theoretical curve reproduces the experimental curve in the same *Q* range. Models with low *R* factors are inferred to represent the average solution structure.

Constrained modelling combines SAXS and SANS data with known crystal structures and sequence information to obtain atomistic models of the global macromolecular structure (Perkins *et al.*, 2011 ▶). Initially, a library of plausible candidate global models is created, which is used to generate theoretical scattering curves for comparison with the SAS data. For this, existing crystal structures alongside homology modelling techniques (Venselaar *et al.*, 2010 ▶) are combined with simulation methodologies such as molecular dynamics or Monte Carlo simulations to produce structurally varied, atomistic, candidate global models of the target macromolecule. Examples of these methods used in classic *SCT* include *DISCOVER3* in *INSIGHT 98* (Accelrys) and the *TorsionKick* function in *Discovery Studio* (Accelrys) (Boehm *et al.*, 1999 ▶; Khan *et al.*, 2010 ▶). From this point, the constrained scattering modelling corresponds to the tools provided by *SCT* (Fig. 2 ▶). First, a grid transformation produces coarse-grained sphere models from the original atomistic structures. Second, the Debye equation is used to calculate a theoretical scattering curve from each sphere model. For modelling SAXS data, a hydration monolayer is added to the sphere model before the scattering curve is calculated. For modelling SANS data, a beam-smearing correction is applied to the theoretical curve. Third, the two curves are compared to see if the calculated curve reproduces what is observed experimentally in the same *Q* range. A quantitative measure of the agreement between the two curves is required. Two quantities are widely used in the literature, namely the *R* factor and 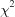. The former is employed within *SCT*. Models showing good curve fits are accepted as potentially representative of the average solution structure. Accepted models can also be filtered from comparisons of 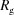 and 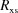 values calculated from Guinier analyses of the theoretical and experimental curves. Depending on the method used to generate the structures it may also be necessary to rule out models where atomic overlap is too high. *SCT* computes the theoretical volume from the protein sequence in order to exclude unphysical overlapping models.

## 3. Algorithms used in *SCT*   

Here, we describe each stage of constrained modelling employed by *SCT*, followed by the programs for each task and the algorithms they use. Table 1 ▶ summarizes the programs used for the various tasks in the process in both the classic and modern versions of *SCT*. At the heart of the constrained modelling process is the computation of a theoretical scattering curve from a protein structural model. Whilst the scattering curve can be calculated from an atomistic structure, this has traditionally been too computationally expensive, although atomistic approaches may become commonplace with improvements in hardware, especially through the availability of modern general-purpose computing on graphics processing units. The widely used alternative employed by *SCT* is to reconstruct the initial atomistic model as a coarse-grained structure using homogeneous, identical spheres of diameter less than the resolution of the scattering experiment. Improvements in the computational resources available usually lead to enhanced conformational sampling and consequently an increase in the number of coarse-grained structures for which curves must be calculated. The Debye equation adapted to spheres is used to calculate 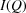 from the sphere model. We describe first how the sphere models are generated and adapted for analysis of SAXS data (in which the hydration layer is observable) and then our implementation of the Debye equation calculation. The modern Python version of *SCT* calls the original Fortran code to perform the scattering calculations, both to increase its speed and to preserve its previously validated method (Smith *et al.*, 1990 ▶; Perkins *et al.*, 1993 ▶; Ashton *et al.*, 1997 ▶).

### 3.1. Generation of sphere models   

**Figure 3:**
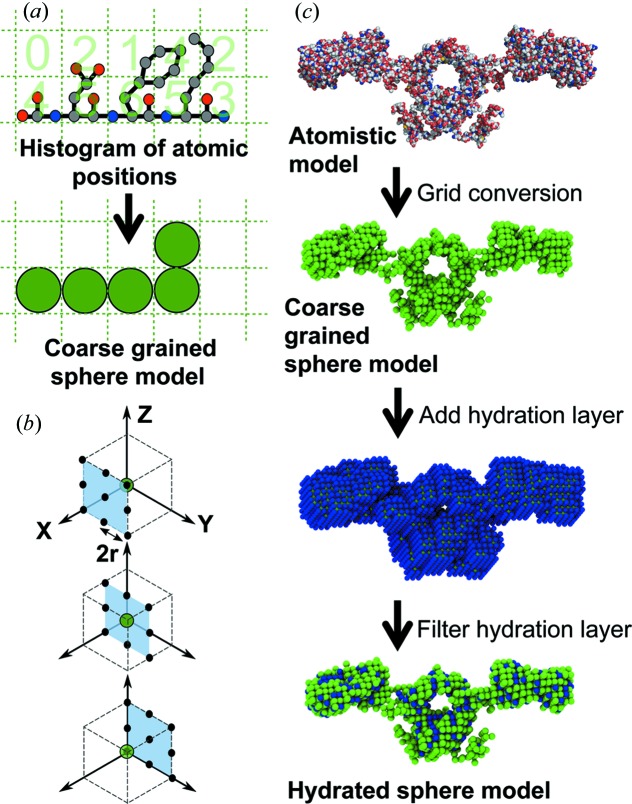
Grid transformation algorithms in *SCT*. (*a*) A two-dimensional schematic of the grid conversion shows how coarse-grained sphere models are derived from atomistic structures. A grid of equal divisions is created that contains all atoms within the input structure. If more than a specified cutoff number of atoms is found within a division, a ‘sphere’ is added to the final model with a radius of half the grid box width. This algorithm is applied in three dimensions to create sphere models from atomistic structures. (*b*) This schematic shows how up to 26 hydration spheres as required are added to each existing sphere in the ‘dry’ model to produce a hydrated sphere model. Hydration spheres are located on the corners and mid-points of the sides of a cube, with a dimension of four times the sphere radius (*r*). The original sphere is shown in green, with the hydration locations in black. (*c*) A hydration layer is required when modelling X-ray scattering data. The hydration layer of water molecules at the surface is added by surrounding each green sphere in the coarse-grained sphere model of the dry protein (top view) with blue hydration spheres of the same radius as shown (middle view). Overlapping and excess blue hydration spheres are subsequently filtered out to match the hydrated volume calculated from the macromolecular sequence, as shown at the bottom.

The generation of a sphere model from an atomistic structure is conceptually simple. Starting from the *x*, *y*, *z* coordinates of the original atomistic model, a histogram of the number of atoms is constructed on a three-dimensional grid with equally sized divisions (grid boxes). Those boxes containing more than a specified cutoff number of atoms are represented by a sphere for the sphere model with the coordinates of the centre of the box and a radius of half the box width. For the *SCT* studies in Supplementary Table S1, the cutoff is usually set to 4. This process is shown schematically in two dimensions in Fig. 3 ▶(*a*). The width of the box (and consequently the radius of the spheres in the final model) is chosen to reproduce the correct protein volume calculated using the *sluv2.py* program of *SCT* (the classic *sluv* program is also included in *SCT* for comparison). The volume of the sphere model is measured by summing the volume of the component spheres.

*sluv2.py* calculates properties including volume, partial specific volume, scattering densities and absorption coefficient of a protein, glycoprotein or carbohydrate from their sequence. For constrained modelling, the most important is the protein volume. This is calculated from the sum of the unhydrated residue volumes (Perkins, 1986 ▶, 2001 ▶). While several volumes are provided using different parameter sets (all of which can be examined in the *aa_volumes.yml* file within the *SCT* distribution), only the consensus unhydrated crystal structure values are used for modelling (Table 2 ▶). *sluv2.py* offers four output methods, namely ‘classic’ (which mimics the output of the original *sluv*), ‘model’, ‘AUC’ and ‘project’. The data contained in the latter three methods are summarized in Table 3 ▶, and a full list of the data output for the classic output is provided in the supporting information.

The sequence can be read in three ways, each of which has different advantages and limitations. These are directly from a PDB file, a FASTA file or a YAML file. A PDB file sequence is read from the ATOM and HETATM records, so any missing residues are not incorporated into the model. FASTA files contain only protein residues. YAML (yet another markup language; http://www.yaml.org/) is a widely used markup language designed to store data in a human-readable and editable format. The organization of the sequence data in the YAML file is proprietary but the format is simple; key, value pairs of three-letter residue codes and frequencies separated by colons (Supplementary Figure S1). Furthermore, residue frequencies can be obtained in YAML format from either FASTA or PDB files using the program *sct_get_sequence.py* provided in the *SCT* package. For example, this makes it straightforward to add glycan residues to a protein sequence in a FASTA file.

### 3.2. Hydration of sphere models   

SAXS reveals the hydrated dimensions of the macromolecule because the hydration shell water has a higher electron density than that of bulk water. This hydration shell is well represented as a monolayer of water molecules that forms a well defined hydrogen-bond arrangement with the protein surface, leading to a shell volume of 0.0245 nm^3^ per bound H_2_O molecule. In contrast, hydrogen bonds continuously break and re-form in bulk water, leading to a volume of 0.0299 nm^3^ per H_2_O molecule. Approximately 0.3 g of water binds per gram of protein or glycoprotein (Perkins, 1986 ▶, 2001 ▶), making macromolecular structures appear bigger when measured by SAXS compared to SANS in ^2^H_2_O buffers. Consequently, SAXS modelling needs to include this hydration layer (Fig. 3 ▶ *b*). *SCT* employs a four-step hydration algorithm for the sphere model in order to reach the correct hydrated volume predicted by *sluv*, using the sequence and the volume of bound water equivalent to 0.3 times the protein or glycoprotein molecular mass (Ashton *et al.*, 1997 ▶):

Step 1. For each sphere in the unhydrated model, add 26 spheres in positions located on the corners and mid-points of the sides of a cube with a dimension of four times the sphere radius and centred on the original sphere (Fig. 3 ▶ *c*).

Step 2. Filter out excess spheres by using a grid conversion similar to that used to create the original unhydrated sphere model from the atomistic structure. As large numbers of spheres are added in Step 1, a high cutoff is used at this stage (typically 10–12 spheres per grid box).

Step 3. The spheres from the original unhydrated model are added back to the results of Step 2. This is done because some extended structures may be lost in the filtering process of Step 2.

Step 4. A final grid conversion with a cutoff of one sphere per grid box is used to filter out any remaining overlapping spheres.

In the classic version of *SCT* the *hypro* program only performs Step 1 of this process. The other steps are incorporated in the *do_curve.sh* script, which runs the whole constrained modelling workflow. For most of the 77 structures (Supplementary Table S1), application of *SCT* involved the selection of a single extended model of the target macromolecule, then applying the above procedure repeatedly using the same cube side chosen for the original dry sphere model, but varying the cutoff used in Step 2. The Python version of *SCT* provides the program *optimize_model_params.py*. This automatically optimizes the cube side and hydration cutoff values to match the theoretical volume derived from the input protein sequence.

### 3.3. Scattering curve calculation using the Debye equation   

The Debye equation relates the spatial distribution of spheres to the scattered intensity as a function of *Q*. When adapted to small spheres, a histogram of the distances *d* between all spheres is constructed. In classic *SCT* the histogram is generated from 400 equally sized bins using a bin width defined by the user. The modern version of *SCT* defines the bin width from the maximum and minimum pair distances between the spheres. Should the user select the minimum and maximum values of the distance histogram to coincide with those found in the structural models, the classic and modern procedures are identical. Once this histogram has been calculated, the 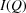 curve as a function of *Q* is obtained from the Debye equation:
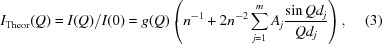
where 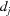 is the distance between spheres represented by the *j*th histogram bin, 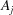 the number of distances that fall into bin *j*, *m* the number of bins in the histogram and *n* the number of spheres in the model. The squared form factor 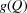 is given by
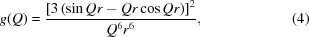
where *r* is the radius of the spheres in the model and is almost unchanged in the *Q* range of interest.

### 3.4. Wavelength spread, beam divergence and incoherent scattering corrections   

Instrumental effects that systematically alter results away from their idealized form are significant in SANS, which is a flux-limited technique, unlike SAXS when this is performed at synchrotron sources. SAXS measurements with laboratory benchtop slit-geometry X-ray instruments are outside the scope of the present *SCT* modelling, because *SCT* was developed for applications at large multi-user X-ray and neutron facilities. In SANS, the main sources of error in the curves come from finite-beam angular divergence (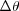), wavelength spread (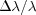) and finite detector resolution (Mildner & Carpenter, 1987 ▶; Barker & Pedersen, 1995 ▶). These effects ‘smear’ the neutron scattering profile, reducing the definition of sharp features, which can significantly impact further analyses. Experimentally, the optimization of the resolution (decreases in 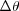 and 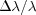) results in reduced scattered intensities; by reducing the resolution, increased SANS intensities are obtained. To account for these effects in SANS modelling, *SCT* enables the user to apply a smearing function to the theoretical curve before comparison with experiment (Perkins & Weiss, 1983 ▶). This is achieved by convoluting the scattering curve with a Gaussian scattering function 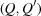:
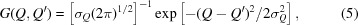
where 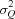 is given by
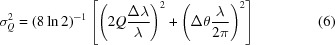
and the 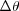 and 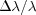 parameters are user specified and tailored to the SANS instrument. While a Gaussian function has proved to be adequate for many earlier *SCT* analyses, more recently developed analyses suggest that other functions such as triangular profiles are more accurate for SANS smearing corrections.

Occasionally, correction is required for the incoherent scattering proton content of a sample measured in heavy water, in which the experimental 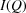 intensities are higher than those calculated at large *Q* values. The correction arises either from the non-exchangeable proton content of the sample at higher concentrations of several mg ml^−1^ or from a minor residual proton content in an incompletely dialysed heavy water buffer. These corrections are typically 0.5–1.5% of the *I*(0) value and are applied as a flat baseline to the theoretical 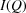 intensities after the curve fitting is completed.

### 3.5. Curve comparison   

The final stage of constrained modelling compares the theoretical scattering curve with the experimental one. In *SCT*, the theoretical 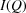 values are matched to the experimental 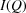 values by taking the theoretical 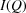 value corresponding to the closest *Q* value seen experimentally. This procedure permitted comparison of the same theoretical curve with multiple experimental data sets, including those from different sessions. After this, the *R* factor is computed, by analogy with crystallography, using the formula
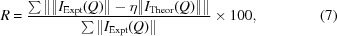
where η is a scaling factor used to match the theoretical curve to the experimental 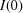. The *R* factor is expressed as a percentage, with lower values representing better fits. An iterative search to minimize the *R* factor is used to determine η. Graphs of the *R* factor *versus R*_g_ values are of great utility in assessing the progression of a modelling fit analysis.

## 4. Application of *SCT*   

All the tools in the classic version of *SCT* are command line utilities which prompt the user directly for input (with the exception of *aps*, which reads sphere models from standard input). A more consistent interface is provided by modern *SCT*, with a series of standardized command line flags passed to the various scripts, and the parameters for analysis and model generation contained in YAML files. The flags for each script are found by running the script with no inputs chosen with the --help flag. The YAML input file format is described in Supplementary Figure S1 and the input parameters are described in Table 4 ▶. The programs used to perform each of the above steps in the constrained modelling workflow are described in Table 1 ▶. Full documentation for all scripts of the *SCT* package is included with the distribution and is available at http://dww100.github.io/sct. A tutorial is also provided with the code and on the web site.

### 4.1. Workflows   

The analysis of thousands of structures requires scripts in both versions of *SCT* to automate the entire process. In the classic version, a bash script *do_curve.sh* is provided, but this requires extensive user editing to ensure that the correct inputs are passed into the individual programs. In the Python version of *SCT*, a single YAML parameter file (Supplementary Figure S2) and the directory containing the input models (in PDB format) are the only inputs required for the *sct_pdb_analysis.py* program to execute the constrained modelling analysis workflow.

Prior to running *sct_pdb_analysis.py*, the sphere model parameters are optimized to reproduce the theoretical volume of the target protein. The *optimize_model_params.py* script automates this process. It takes a PDB structure and optionally a separate target sequence and obtains the cube side value which best reproduces the unhydrated protein volume, together with a list of hydration cutoff values in order to enable the user to determine the optimal hydration cutoff to recreate the hydrated volume. These values should then be added to the YAML file. A typical command line used to run the optimization procedure isoptimize_model_params.py -p params.yml -i pdbs/extended.pdb -o optimized_cutoffs.dat -s sequence.faswhere params.yml is a YAML parameter file, pdbs/extended.pdb is the path to a PDB file with no overlapping atoms, optimized_cutoffs.dat is the output file and sequence.fas is a FASTA file containing the full sequence for the target protein. A sequence file is used when a small number of residues are missing from the structure to ensure that a sphere model of the correct volume is constructed. Nonetheless, it is preferable to complete the initial input model using molecular modelling software, because the extra volume is otherwise unlikely to be correctly distributed in the generated sphere models.

The main *SCT* workflow is performed by the script *sct_pdb_analysis.py*. This takes the path to a directory of PDB files, and to the experimental data for comparison, and inputs (alongside the YAML parameter file) and returns curves and sphere models in PDB format for each atomistic input along with two output data files, which are formatted as tab-separated columns. The first output lists the input experimental data files and the 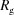 and 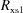 values calculated for them. The second output provides the comparison of the PDB structures with each curve. Examples from the *SCT* tutorial are supplied in the supporting information. The 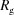 and 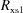 values are calculated for each theoretical curve, together with the 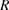 factor comparing it with each experimental curve. The parameters for the sphere and curve fitting and comparison, such as *Q* ranges, are supplied in the input YAML file. A typical command to run the full workflow issct_pdb_analysis.py -p params.yml -i pdbs/-x expt/x.dat -n expt/n.dat -o sct_outputwhere params.yml is a YAML parameter file, pdbs is a directory containing the atomistic model PDB files, expt/x.dat and expt/n.dat are the experimental SAXS and SANS curves, respectively, and sct_output is the path into which the calculated output will be placed. The generated sphere models and curves are placed in directories <method>/models and <method>/curves under sct_output, where <method> is either xray or neutron.

**Figure 4:**
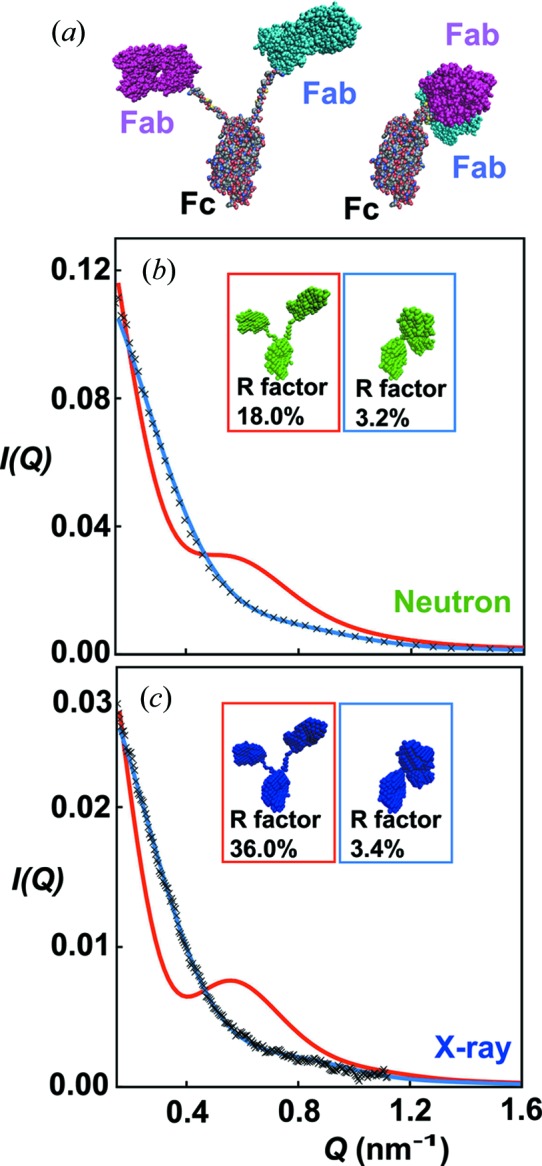
Comparison of neutron and X-ray scattering data with models of human immunoglobulin IgG4 composed of Fab and Fc regions (Rayner *et al.*, 2014 ▶). (*a*) Atomistic structures of an extended asymmetric (left) and a compact asymmetric (right) IgG4 model. In these, the Fc region is viewed in a similar orientation. (*b*), (*c)* Comparisons of the calculated scattering curves for sphere models generated by *SCT* from the atomistic models with neutron and X-ray experimental data, respectively. The *Q* range is depicted from 0.2 to 1.6 nm^−1^. In both cases, the compact asymmetric model (blue curve: PDB code 4pto) gives good fits to the experimental data, whereas the extended model (red curve) does not.

The application of the modern *SCT* workflow is illustrated for two human immunoglobulin IgG4 models (Rayner *et al.*, 2014 ▶) (Fig. 4 ▶). The two conformations are significantly different, one being extended with the two Fab regions distal from one another, and the other being highly asymmetric with both Fab regions packed together. The sphere models provide good visual representations of the atomistic conformations. The scattering curve from the compact model reproduces the SANS and SAXS experimental curves better than that of the extended model.

### 4.2. Python package   

All of the functionality used to build the scripts of the *SCT* package is accessible *via* a Python package, allowing advanced users to create their own modified workflows. The Python package contains five modules termed *seq*, *pdb*, *sphere*, *curve* and *param*. As the names of the first four imply, these four are each concerned with the processing of different types of data (sequence, atomistic structures, sphere models and scattering curves, respectively). The *param* module reads and validates the YAML parameter files. The package has three main dependencies, termed *PyYAML* (http://pyyaml.org/wiki/PyYAML), *NumPy* and *SciPy* (both available from http://www.scipy.org/), and utilizes *matplotlib* (http://matplotlib.org/) for graphing. Elements of the classic code are linked using *F2PY*, which is provided with *NumPy*. All of these dependencies are freely available and found in common scientific Python distributions. Once installed the package is loaded usingimport sct

Full technical documentation of the *SCT* package can be found at http://dww100.github.io/sct or generated from the source code using *Epydoc* (http://epydoc.sourceforge.net/).

## 5. Conclusions and outlook   

In this report, we describe, update and publicly release the *SCT* suite, which enables the computation of theoretical scattering curves from atomistic models *via* coarse-grained sphere models and their comparison with experimental SAXS and SANS data. Whilst these tools have a history stretching back several decades, this is the first time they have been described together and released as open source. In addition to the classic Fortran version of *SCT*, we provide a modern, easier-to-use reimplementation in Python that makes calls to this classic validated Fortran code. Our two design goals with the Python version are to maintain the *SCT* algorithms and functionality that have produced 77 structures to date (Supplementary Table S1) and to provide *SCT* with a simpler interface to (i) assist the programs’ application to larger numbers of trial models in more ambitious projects and (ii) allow *SCT* to be integrated into a user’s own workflow. To further this goal, we are incorporating *SCT* as the primary scattering curve calculator in the modelling tool *SASSIE* (Curtis *et al.*, 2012 ▶) in the *CCP-SAS* project. The aim of the *CCP-SAS* project is to provide a suite of open-source simulation and analysis tools for the atomistic modelling of scattering curves within a unified graphical user interface, including a web-based front-end and a back-end based on high-performance computing hardware.

## Supplementary Material

Here we describe the output of the sluv tools, a table of the macromolecular structures previously solved using SCT, and figures to elucidate some of the input formats used by modern SCT.. DOI: 10.1107/S1600576715007062/po5031sup1.pdf

## Figures and Tables

**Table 1:** Tasks involved in the constrained modelling process and the programs within the *SCT* suite that perform them The programs in both the classic (Fortran-based) and modern (Python-based) versions are shown for each task.

Task	Modern	Classic
Volume calculation	*sluv2.py*	*sluv*
Sphere model parameter optimization	*optimize_model_params.py*	
Sphere model creation	*pdb2sphere.py*	*brktos*
Sphere model hydration	*hydrate_spheres.py*	*hypro*
Calculate *R*_g_ from sphere model	*sphere_rg.py*	*aps*
Theoretical scattering calculation	*calculate_curve.py*	*sct*
Calculate *R*_g_ from curve	*sas_curve_analysis.py*	*sctpl*
Curve comparison	*calculate_rfactor.py*	*rfacXN*
Workflow	*sct_pdb_analysis.py*	*do_curve.sh*

**Table 2:** Residue volumes for both amino acids and monosaccharides used by *sluv2.py* in *SCT* to calculate the macromolecular volumes used in constrained modelling (Perkins, 1986 ▶) In the ‘classic’ output option, as well as the output of classic *sluv*, these residue volumes are labelled PER85. The non-agreement of the naming with the 1986 publication is maintained for historical reasons.

Residue name	Residue code	Volume (10^3^nm^3^)
Alanine	ALA	97.1
Arginine	ARG	192.9
Asparagine	ASN	127.4
Aspartic acid	ASP	125.3
Cysteine	CYS	112.4
Glutamine	GLN	147.3
Glutamic acid	GLU	148.0
Glycine	GLY	68.2
Histidine	HIS	158.3
Isoleucine	ILE	170.1
Leucine	LEU	182.8
Lysine	LYS	184.5
Methionine	MET	176.0
Phenylalanine	PHE	203.9
Proline	PRO	129.0
Serine	SER	103.3
Threonine	THR	129.0
Tryptophan	TRP	228.9
Tyrosine	TYR	202.3
Valine	VAL	142.3
Fucose	FUC	160.8
Galactose	GAL	166.8
Glucose	GLC	171.9
Mannose	MAN	170.8
*N*-Acetylglucosamine	NAG	222.0
*N*-Acetylgalactosamine	NGA	232.9
Sialic acid	SIA	326.3

**Table 3:** Data output from the three new output modes introduced in *sluv2.py* All data are included in the ‘classic’ output mode and output from *sluv*.

Output type	Macromolecular molecular weight (10^3^kgmol^1^)	Absorption coefficient	Partial specific volume (nm^3^kg^1^)	Macromolecular volume (10^3^nm^3^)
Model	No	No	No	Yes
AUC	Yes	Yes	Yes	No
Project	Yes	Yes	Yes	Yes

**Table 4:** Explanation of the parameters in the YAML input to the modern version of *SCT* The same parameters are required for 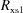 and 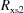, consequently they are both denoted rxs?. The format of the YAML file is shown in Supplementary Figure S2.

Parameter		Type	Meaning
wide	qmin	Float	Minimum *Q* value used in wide-angle plot
wide	qmax	Float	Maximum *Q* value used in wide-angle plot
rg	qmin	Float	Minimum *Q* value used in 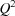 *versus* 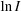 plot (from which 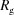 is calculated)
rg	qmax	Float	Maximum *Q* value used in 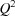 *versus* 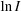 plot (from which 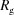 is calculated)
rg	fitmin	Float	Minimum *Q* value used in linear fit of 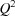 *versus* 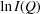 from which 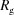 is calculated
rg	fitmax	Float	Maximum *Q* value used in linear fit of 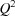 *versus* 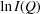 from which 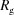 is calculated
rxs?	qmin	Float	Minimum *Q* value to be plotted in the region used to calculate 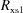 / 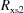
rxs?	qmax	Float	Maximum *Q* value to be plotted in the region used to calculate 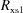 / 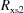
rxs?	fitmin	Float	Minimum *Q* value used in the linear fit from which 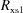 / 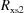 are calculated
rxs?	fitmax	Float	Maximum *Q* value used in the linear fit from which 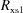 / 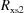 are calculated
sphere	cutoff	Integer	Cutoff of the number of atoms in a grid box over which a sphere is added to a sphere model
sphere	boxside	Float	The length of the side of the grid boxes used in sphere model creation
hydrate	positions	Integer	Number of positions surrounding each atom onto which a ‘hydration sphere’ should be added when creating a hydrated sphere model (see Fig. 1 ▶ *c*)
hydrate	cutoff	Integer	Cutoff used to remove excess hydration spheres when creating a hydrated sphere model
curve	qmax	Float	Maximum *Q* value for the theoretical scattering curve
curve	npoints	Integer	Number of points at which to calculate *I* in the scattering curve (between 0 and qmax)
curve	radbins	Integer	Number of bins in the distance histogram used with the Debye equation
curve	smear	Boolean	Choice of whether to include a smearing correction in the scattering curve
curve	wavelength	Float	Wavelength used in smearing calculation
curve	spread	Float	Wavelength spread 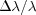 used in smearing calculation
curve	divergence	Float	Beam divergence 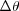 used in smearing calculation
rfac	qmin	Float	Minimum *Q* value used to compare curves (calculate the 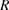 factor)
rfac	qmax	Float	Maximum *Q* value used to compare curves (calculate the 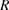 factor)

## Footnotes

1 For literature related to these structures see Abe *et al.* (2010 ▶), Almogren *et al.* (2006 ▶), Aslam & Perkins (2001 ▶), Aslam *et al.* (2003 ▶), Boehm & Perkins (2000 ▶), Boehm *et al.* (1999 ▶), Bonner *et al.* (2007 ▶, 2008 ▶, 2009*a* ▶,*b* ▶), Fernando *et al.* (2007 ▶), Furtado *et al.* (2004 ▶, 2008 ▶), Gilbert *et al.* (2005 ▶), Gilbert, Aslam *et al.* (2006 ▶), Gilbert, Asokan *et al.* (2006 ▶), Hu *et al.* (2005 ▶), Khan *et al.* (2010 ▶, 2011 ▶), Khan, Gor *et al.* (2013 ▶), Khan, Fung *et al.* (2013 ▶), Li *et al.* (2010 ▶, 2012 ▶), Nan *et al.* (2010 ▶), Miller *et al.* (2012 ▶), Okemefuna *et al.* (2008 ▶, 2009 ▶, 2010 ▶), Perkins *et al.* (1991 ▶), Rayner *et al.* (2013 ▶, 2014 ▶, 2015 ▶), Rodriguez *et al.* (2015 ▶) and Sun *et al.* (2004 ▶, 2005 ▶).
