# Supplementary material for: SCT: a suite of programs for comparing atomistic models with small-angle scattering data
Source: J Appl Crystallogr. 2015 May 9;48(Pt 3):953–61. doi: 10.1107/S1600576715007062 (PMC4453981; doi:10.1107/S1600576715007062)
Supplement: Supplementary file 1 [file j-48-00953-sup1.pdf]

SUPPLEMENTARY INFORMATION

SCT: A Suite of Programs for Comparing Atomistic Models to Small Angle Scattering Data

Here we describe the output of the *sluv* tools, a table of the macromolecular structures previously solved using SCT, and figures to elucidate some of the input formats used by modern SCT. A separate file SCT_workflow_example.zip is available and contains the output from the tutorial workflow discussed in the main paper. The data used to obtain the results are distributed alongside SCT as the ‘modern tutorial’.

Sluv Output

The output of sluv (abbreviation for “scattering length per unit volume) is made up of 9 sections. The first section is a table providing a by residue type breakdown of the volume, molecular weight, number of electrons and scattering length (in D_2O and H_2O) of the glycoprotein. The volume is calculated using all 7 datasets listed in (Perkins, 1986). The second section reports summary data for the entire glycoprotein. This lists molecular weight, extinction coefficient, scattering length, number of electrons, partial specific volume, matchpoint and scattering density at the matchpoint. The third section details the hydration of the protein. This is followed by four sections which breakdown the summary data shown for the whole system for different subsets of the structure (protein only, non-polar residues only, polar residues only and carbohydrate alone). The final two sections detail the hydrogens which are exchangeable with deuterium. The first of these is concerned with the peptide backbone hydrogens alone, the second with all hydrogens.

Supplementary Table S1. Seventy-seven solution structures determined using the SCT suite which have been deposited in the Protein Data Bank (PDB) at http://www.rcsb.org/. These correspond to 14 different antibody-related structures (totalling 24), seven different complement-related structures and their fragments (totalling 27), two anionic oligosaccharides (totalling 24) and two miscellaneous studies. From 2010, the coordinate files were curated by the PDB, but were no longer made available on its web site. These curated files were made available in the supporting information of the publications made since 2010.

PDB code	Title	Deposition Date	Publication
1IGA	Human immunoglobulin IgA1 monomer	23 Dec 1998	Boehm *et al.* (1999)
1E07	Human carcinoembryonic antigen	11 Mar 2000	Boehm & Perkins (2000)
1HAQ	Human complement Factor H	06 Apr 2001	Aslam & Perkins (2001)
1NTJ	Rat complement receptor-related gene/protein y (Crry)	30 Jan 2003	Aslam *et al.* (2003)
1NTL	Mouse Crry-Ig chimaeric conjugate	30 Jan 2003	Aslam *et al.* (2003
1R70	Human immunoglobulin IgA2 monomer	17 Oct 2003	Furtado *et al.* (2004)
1W2R	Human complement receptor Type 2 (CR2) SCR-1/2	04 Jun 2004	Gilbert *et al.* (2005)
1W2S	Human CR2 SCR-1/2 and C3d	04 Jun 2004	Gilbert *et al.* (2005)
1W0R	Human properdin dimer	09 Jun 2004	Sun *et al.* (2004)
1W0S	Human properdin trimer	09 Jun 2004	Sun *et al.* (2004)
1ZLG	Extracellular matrix protein anosmin-1	06 May 2005	Hu *et al.* (2005)
1ZVO	Human myeloma immunoglobulin D	02 Jun 2005	Sun *et al.* (2005)
2ATY	Human CR2-Ig chimaeric conjugate	26 Aug 2005	Gilbert *et al.* (2006a)
2ESG	Human myeloma IgA-human serum albumin	26 Oct 2005	Almogren *et al.* (2006)
2GSX	Human CR2 SCR-1/15	27 Apr 2006	Gilbert *et al.* (2006b)
2IC4	Human complement factor H SCR-6/8	12 Sep 2006	Fernando *et al.* (2007)
2OCW	Human secretory component	21 Dec 2006	Bonner *et al.* (2007)
2Q7Z	Human complement receptor Type 1 (CR1) SCR-1/30	08 Jun 2007	Furtado *et al.* (2008)
2QFG	Human complement factor H SCR-1/5	27 Jun 2007	Okemefuna *et al.* (2008)
2QFH	Human complement factor H SCR-16/20	27 Jun 2007	Okemefuna *et al.* (2008)
2QTJ	Human immunoglobulin IgA1 dimer	02 Aug 2007	Bonner *et al.* (2008)
2RCJ	Human immunoglobulin IgM pentamer	20 Sep 2007	Perkins *et al.* (1991)
3CHN	Human immunoglobulin secretory IgA1	10 Mar 2008	Bonner *et al.* (2009a)
3CM9	Human immunoglobulin secretory IgA2	21 Mar 2008	Bonner *et al.* (2009b)
3GAU	Human complement Factor H SCR-1/20 in 50 mM NaCl	18 Feb 2009	Okemefuna *et al.* (2009)
3GAV	Human complement Factor H SCR-1/20 in 137 mM NaCl	18 Feb 2009	Okemefuna *et al.* (2009)
3GAW	Human complement Factor H SCR-1/20 in 250 mM NaCl	18 Feb 2009	Okemefuna *et al.* (2009)
3IRI	Heparin oligosaccharide dp18 *	24 Aug 2009	Khan *et al.* (2010)
3IRJ	Heparin oligosaccharide dp24 *	24 Aug 2009	Khan *et al.* (2010)
3IRK	Heparin oligosaccharide dp30 *	24 Aug 2009	Khan *et al.* (2010)
3IRL	Heparin oligosaccharide dp36 *	24 Aug 2009	Khan *et al.* (2010)
3M7X **	Human-mouse immunoglobulin IgG4 at 1.3 mg/ml	17 Mar 2010	Abe *et al.* (2010)
3M7Y	Human-mouse immunoglobulin IgG4 at 0.98 mg/ml	17 Mar 2010	Abe *et al.* (2010)
3M7Z	Human-mouse immunoglobulin IgG4 at 0.65 mg/ml	17 Mar 2010	Abe *et al.* (2010)
3M80	Human-mouse immunoglobulin IgG4 at 0.3 mg/ml	17 Mar 2010	Abe *et al.* (2010)
3MMQ	Human complement C3u in 137 mM NaCl	20 Apr 2010	Li *et al.* (2010)
3N0J	Homozygous human complement Factor H (Y402/H402)	14 May 2010	Nan *et al.* (2010)
3N8O	Homozygous human complement Factor H (Y402/H402)	28 May 2010	Okemefuna *et al.* (2010)
3N8P	Human complement factor H SCR-8/11	28 May 2010	Okemefuna *et al.* (2010)
3N8Q	Human complement factor H SCR-11/15	28 May 2010	Okemefuna *et al.* (2010)

3QHG	Heparan sulphate oligosaccharide dp6 ***	26 Jan 2011	Khan *et al.* (2011)
3QHH	Heparan sulphate oligosaccharide dp8 ***	26 Jan 2011	Khan *et al.* (2011)
3QHI	Heparan sulphate oligosaccharide dp10 ***	26 Jan 2011	Khan *et al.* (2011)
3QHJ	Heparan sulphate oligosaccharide dp12 ***	26 Jan 2011	Khan *et al.* (2011)
3QHK	Heparan sulphate oligosaccharide dp14 ***	26 Jan 2011	Khan *et al.* (2011)
3QHL	Heparan sulphate oligosaccharide dp16 ***	26 Jan 2011	Khan *et al.* (2011)
3TKO	Complement therapeutic inhibitor TT30	07 Sep 2011	Li *et al.* (2012)
3UJU	Human complement MBL monomer	08 Nov 2011	Miller *et al.* (2012)
3UJV	Human complement MBL dimer	08 Nov 2011	Miller *et al.* (2012)
3UJW	Human complement MBL trimer	08 Nov 2011	Miller *et al.* (2012)
3UJX	Human complement MBL tetramer	08 Nov 2011	Miller *et al.* (2012)
4GDQ	Rabbit immunoglobulin IgG in 137 mM NaCl	01 Aug 2012	Rayner *et al.* (2013)
4GDR	Rabbit immunoglobulin IgG in 250 mM NaCl	01 Aug 2012	Rayner *et al.* (2013)
4GDS	Rabbit immunoglobulin IgG in 137 mM NaCl (^2H_2O)	01 Aug 2012	Rayner *et al.* (2013)
4J8H	Heparin oligosaccharide dp18 *	14 Feb 2013	Khan *et al.* (2013a)
4J8I	Heparin oligosaccharide dp24 *	14 Feb 2013	Khan *et al.* (2013a)
4J8J	Heparin oligosaccharide dp30 *	14 Feb 2013	Khan *et al.* (2013a)
4J8K	Heparin oligosaccharide dp36 *	14 Feb 2013	Khan *et al.* (2013a)
4KHC	Heparan sulphate oligosaccharide dp6 ***	30 Apr 2013	Khan *et al.* (2013b)
4KHD	Heparan sulphate oligosaccharide dp8 ***	30 Apr 2013	Khan *et al.* (2013b)
4KHE	Heparan sulphate oligosaccharide dp10 ***	30 Apr 2013	Khan *et al.* (2013b)
4KHF	Heparan sulphate oligosaccharide dp12 ***	30 Apr 2013	Khan *et al.* (2013b)
4KHG	Heparan sulphate oligosaccharide dp14 ***	30 Apr 2013	Khan *et al.* (2013b)
4KHH	Heparan sulphate oligosaccharide dp16 ***	30 Apr 2013	Khan *et al.* (2013b)
4KHI	Heparan sulphate oligosaccharide dp18 (extended) ***	30 Apr 2013	Khan *et al.* (2013b)
4KHJ	Heparan sulphate oligosaccharide dp18 (bent) ***	30 Apr 2013	Khan *et al.* (2013b)
4KHK	Heparan sulphate oligosaccharide dp24 (extended) ***	30 Apr 2013	Khan *et al.* (2013b)
4KHL	Heparan sulphate oligosaccharide dp24 (bent) ***	30 Apr 2013	Khan *et al.* (2013b)
4MRJ	Human complement C3b in 50 mM NaCl	17 Sep 2013	Rodriguez *et al.* (2015)
4MRK	Human complement C3b in 137 mM NaCl	17 Sep 2013	Rodriguez *et al.* (2015)
4MRL	Human complement C3u in 50 mM NaCl	17 Sep 2013	Rodriguez *et al.* (2015)
4PTO	Human immunoglobulin IgG4 (Ser222) by X-rays	11 Mar 2014	Rayner *et al.* (2014)
4PTQ	Human immunoglobulin IgG4 (Ser222) by neutrons	11 Mar 2014	Rayner *et al.* (2014)
4PTR	Human immunoglobulin IgG4 (Pro222) by X-rays	11 Mar 2014	Rayner *et al.* (2014)
4QOU	Human immunoglobulin IgG1 6a by X-rays	20 Jun 2014	Rayner *et al.* (2015)
4QOV	Human immunoglobulin IgG1 19a by X-rays	20 Jun 2014	Rayner *et al.* (2015)
4QOW	Human immunoglobulin IgG1 6a by neutrons	20 Jun 2014	Rayner *et al.* (2015)

* The original heparin coordinates were corrected in Khan *et al.* (2013a)

** Coordinate files from this point onward are published in the Supplementary Material of the associated publications.

*** The original heparan sulphate coordinates were corrected in Khan *et al.* (2013b)

```
ARG:  52
ASN:  34
ASP:  50
GLN:  66
GLU:  66
HIS:  22
LYS:  66
SER:  170
THR:  130
(etc . . .)
```

Supplementary Figure S1

Format of the YAML file used by *sluv2.py* to contain the residue frequencies of a system under investigation. The two columns are the three letter residue codes and the frequency of their occurrence in the structure under investigation. The valid residue names are listed in Table 3 of the main text.

```yaml
wide:
    qmin: 0.00
    qmin: 0.30
rg:
    qmin: 0.00
    qmax: 0.05
    fitmin: 0.013
    fitmax: 0.028
rxs1:
    qmin: 0.00
    qmin: 0.07
    fitmin: 0.030
    fitmax: 0.051
rxs2:
    qmin: 0.00
    qmin: 0.15
    fitmin: 0.056
    fitmax: 0.14
sphere:
    cutoff: 4
    boxside: 5.19
hydrate:
    positions: 27
    cutoff: 11
curve:
    qmax: 0.25
    npoints: 100
    radbins: 400
    smear: True
    wavelength: 6.0
    spread: 0.1
    divergence: 0.016
rfac:
    qmin: 0.013
    qmax: 0.2
```


Supplementary Figure S2

Format of the YAML input file used by the modern version of SCT. The coloured rectangles are used to visually indicate related sets of parameters; blue for graph plotting and fitting ranges, green for sphere model creation, purple for scattering curve calculation and orange for curve comparison

References for Supplementary Table S1

Abe, Y., Gor, J., Bracewell, D. G., Perkins, S. J. & Dalby, P. A. (2010). *Biochem. J.* **432**, 101-111.

Almogren, A., Furtado, P. B., Sun, Z., Perkins, S. J. & Kerr, M. A. (2006). *J. Mol. Biol.* **356**, 413-431.

Aslam, M. & Perkins, S. J. (2001). *J. Mol. Biol.* **309**, 1117-1138.

Aslam, M., Guthridge, J. M., Hack, B. K., Quigg, R. J., Holers, V. M. & Perkins, S. J. (2003). *J. Mol. Biol.* **329**, 525 – 550.

Boehm, M. K. & Perkins, S. J. (2000). *FEBS Letters* **475**, 11-16.

Boehm, M. K., Woof, J. M., Kerr, M. A. & Perkins, S. J. (1999). *J. Mol. Biol.* **286**, 1421-1447.

Bonner, A., Perrier, C., Corthésy, B. & Perkins, S. J. (2007). *J. Biol. Chem.* **282,** 16969 - 16980.

Bonner, A., Furtado, P. B., Almogren, A., Kerr, M. A. & Perkins, S. J. (2008). *J. Immunol.* **180**, 1008-1018.

Bonner, A., Almogren, A., Furtado, P. B., Kerr, M. A. & Perkins, S. J. (2009a). *Mucosal Immunology,* **2**, 74-84.

Bonner, A., Almogren, A., Furtado, P. B., Kerr, M. A. & Perkins, S. J. (2009b). *J. Biol. Chem.* **284**, 5077-5087.

Fernando, A. N., Furtado, P. B., Clark, S. J., Gilbert, H. E., Day, A. J., Sim, R. B. & Perkins, S. J. (2007). *J Mol Biol*. **368**, 564-581.

Furtado, P. B., Whitty, P.W., Robertson, A., Eaton, J. T., Almogren, A., Kerr, M.A., Woof, J.M. & Perkins, S. J. (2004). *J. Mol. Biol.* **338**, 921-941.

Furtado, P. B., Huang, C. Y., Ihyembe, D., Hammond, R. A., Marsh, H. C. & Perkins, S. J. (2008). *J. Mol. Biol.* **375**, 102-118.

Gilbert, H. E., Eaton, J. T., Hannan, J. P., Holers, V. M., and Perkins, S. J. (2005). *J. Mol. Biol.* **346**, 859-873.

Gilbert, H. E., Aslam, M., Guthridge, J. M., Holers, V. M. & Perkins, S. J. (2006a). *J. Mol. Biol.* **356**, 397-412.

Gilbert, H. E., Asokan, R., Holers, V. M. & Perkins, S. J. (2006b). *J. Mol. Biol.* **362**, 1132-1147.

Hu, Y., Sun, Z., Eaton, J. T., Bouloux, P. M. G. & Perkins, S. J. (2005). *J. Mol. Biol.* **350**, 553-570.

Khan, S., Gor, J., Mulloy, B. & Perkins, S. J. (2010). *J. Mol. Biol.* **395**, 504-521.

Khan, S., Rodriguez, E., Patel, R., Gor, J., Mulloy, B. & Perkins, S. J. (2011). *J. Biol. Chem.* **286**, 24842–24854 (withdrawn).

Khan, S., Gor, J., Mulloy, B. & Perkins, S. J. (2013a). *J. Mol. Biol.* **425**, 1847. *Corrigendum.*

Khan, S., Fung, K.-W., Rodriguez, E., Patel, R., Gor, J., Mulloy, B. & Perkins, S. J. (2013b). *J. Biol. Chem.* **288**, 27737-27751.

Li, K., Gor, J. & Perkins, S. J. (2010). *Biochem. J.* **431**, 63-72.

Li, K., Gor, J., Holers, V. M., Storek, M. J. & Perkins, S. J. (2012). *J. Mol. Biol.* **418**, 248-263.

Nan, R., Ward, G., Gavigan, L., Miller, A., Gor, J., McKay, A. R., Lengyel, I. & Perkins, S. J. (2010). Molec. Immunol. **47**, 2263-2263 (abstract).

Miller, A., Phillips, A., Gor, J., Wallis, R. & Perkins, S. J. (2012). *J. Biol. Chem.* **287**, 3930-3945.

Okemefuna, A. I., Gilbert, H. E., Griggs, K. M., Ormsby, R. J., Gordon, D. L. & Perkins, S. J. (2008). *J. Mol. Biol*. **375**, 80-101.

Okemefuna, A. I., Nan, R., Gor, J. & Perkins, S. J. (2009). *J. Mol. Biol*. **391**, 98-118.

Okemefuna, A. I., Gor, J., Sadlon, T., Adamson, P., Gordon, D. L. & Perkins, S. J. (2010). Unpublished modelling.

Perkins, S. J., Nealis, A. S., Sutton, B. J. & Feinstein, A. (1991). *J. Mol. Biol.* **221**, 1345-1366.

Rayner, L. E., Kadkhodayi-Kholghi, N., Heenan, R. K., Gor, J., Dalby, P. A. & Perkins S. J. (2013). *J. Mol. Biol.* **425**, 506-523.

Rayner, L. E., Hui, G. K., Gor, J., Heenan, R. K. Dalby, P. A. & Perkins S. J. (2014). *J. Biol. Chem.* **289**, 20740-20756.

Rayner, L. E., Hui, G. K., Gor, J., Heenan, R. K., Dalby, P. A. & Perkins S. J. (2015). *J. Biol. Chem.* **290**, 8420-8438.

Rodriguez, E., Nan, R., Li., K., Gor, J. & Perkins, S. J. (2015). *J. Biol. Chem.* **290**, 2334-2350.

Sun, Z., Reid, K. B. M. and Perkins, S. J. (2004). *J. Mol. Biol.* **343**, 1327-1343.

Sun, Z., Almogren, A., Furtado, P. B., Chowdhury, B., Kerr, M. A. & Perkins, S. J. (2005). *J. Mol. Biol.* **353**, 155-173.
